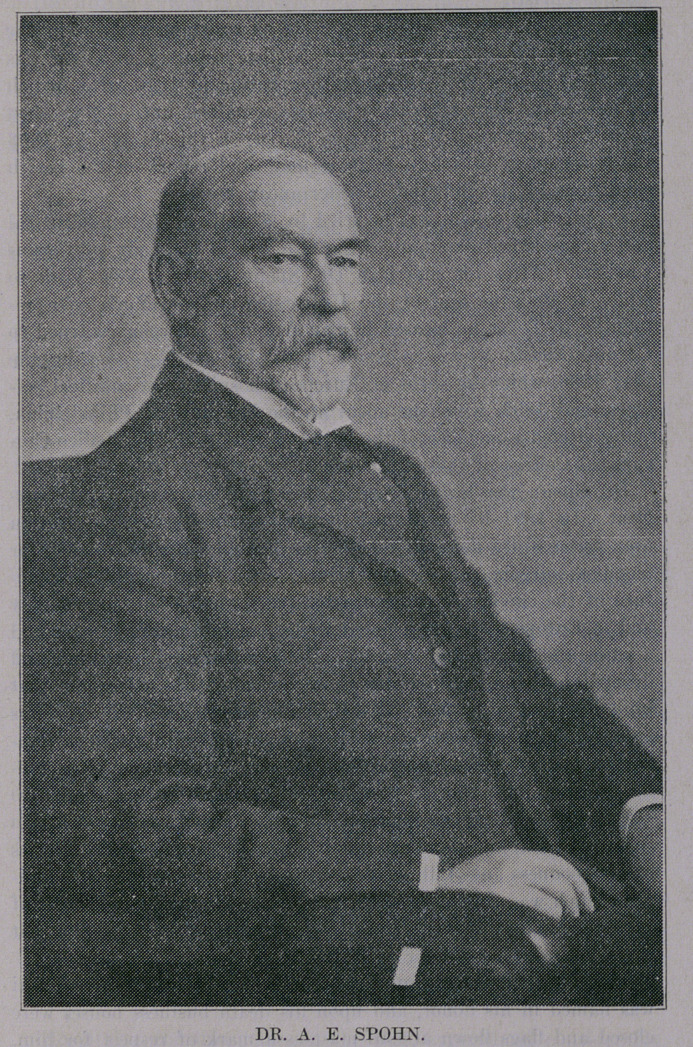# Dr. A. E. Spohn

**Published:** 1913-06

**Authors:** R. H. L. Bibb


### Dr. A. E. Spohn.

After an active and a successful professional life of nearly a half century, Dr. Arthur Edward Spohn died at his home in Corpus Christi, Texas, May 2, 1913, aged 68 years.

Dr. Spohn was born in Ancaster, Canada, April 27, 1845. He was educated in Borria High School and McGill University, and graduated from Ann Arbor, Michigan, Bellevue Hospital Medical College, and Long Island College Hospital, New York, and was assistant professor of surgical anatomy in the latter college in 1867-68. McGill University awarded him the senior prize for practical anatomy in 1865. He was a member of King's County Medical Society, New York; president of the Nueces County Medical Society, member of the American Medical Association, the American Association for the Advancement of Science, the United States Association of Military Surgeons, of the State Medical Association of Texas, the Pan-American Medical Congress, the International Association for the Study of Tuberculosis and of the Central Texas Medical Association. He was chief surgeon of the St. Louis,

DR. A. E. SPOHN.

Brownsville and Mexico Railroad, and for the past fifteen years was connected with the United States Marine Hospital Service at Corpus Christi.

Dr. Spohn came to Texas in 1868 as assistant surgeon in the United States Army and served in that capacity until he was placed in charge of the military quarantine in 1870, after which he located in Mier, Mexico, where he remained until 1872, when he located permanently in Corpus Christi in this State. In 1903 the government sent him to investigate and report on the health conditions of the different Mediterranean seaports.

In 1876 Dr. Spohn was married to Miss Sarah I., the beautiful and accomplished daughter of Captain Mifflin Kennedy, of Corpus Christi. After his marriage Dr. Spohn went to New York to study at Bellevue and at the University of New York. On returning to Texas he located in San Antonio, but soon returned to Corpus Christi, where he resided until his death (excepting the year 1888, which he spent at European hospitals and medical colleges, and 1892, which he spent at the hospitals of Philadelphia, being elected a member of the board of censors of the Medico-Chirurgical College of that city in 1894).

Dr. Spohn was a dexterous, a courageous and a successful surgeon. In 1891 he performed the first Porro-Cæsarian operation ever successfully done in the United States. His tourniquet for bloodless surgery and his apparatus for the treatment of the fracture of the clavicle have been extensively adopted in this country and in Europe. He was a fluent writer, an interesting speaker and a calm and convincing debater. He was an indulgent, kind and devoted husband, with whom those delicate little attentions that bind the soul of wife to husband were never omitted. He was a law-abiding, a just and an upright citizen. He was a philanthropist whose warm and responsive heart throbbed with the tenderest emotions wherever there was a wound to heal or a pain to ease. No one, however humble, however poor, ever appealed to Spohn in vain, and there are hundreds in Texas today, beneficiaries of his goodness and skill, whose gratitude, coupled with that happiness which was always his whenever he could minister to the ills of his fellow man, has been his only recompense, and who mourn that their generous benefactor is no more.

The beautiful sanatorium built by the people of Corpus Christi was named in his honor, and upon his death business houses were closed and flags flown at half-mast as a mark of respect for him. An afflicted wife survives him. R. H. L. Bibb.

## Figures and Tables

**Figure f1:**